# Mex3a expression and survival analysis of bladder urothelial carcinoma

**DOI:** 10.18632/oncotarget.18399

**Published:** 2017-06-07

**Authors:** Jing-wen Shi, Ying Huang

**Affiliations:** ^1^ Department of Ultrasound, Shengjing Hospital of China Medical University, 110004, Shenyang, China

**Keywords:** mex3a, bladder urothelial carcinoma, overall survival, TCGA, pathology

## Abstract

**Objective:**

Bladder urothelial carcinoma is a common tumor in humans and a multifactorial disease. The gene *mex3a* is associated with tumor formation and may promote cell proliferation and migration. Therefore, this study aimed to determine the relationship between *mex3a* and bladder urothelial carcinoma.

**Methods:**

The clinical and RNA sequencing expression data in patients with bladder urothelial carcinoma were downloaded from the The Cancer Genome Atlas data portal. A total of 412 bladder urothelial carcinoma samples were available in the database, for which the clinical information was acquired, of which 412 are RNA sequencing samples with a total of 19 paired samples. Univariate and multivariate Cox analyses and univariate logistic regression analysis were conducted using the software SPSS version 22.0 and *P*<0.05 was considered statistically significant.

**Results:**

The results of the independent *t*-test of 19 paired samples indicated that the expression level of *mex3a* was significantly higher in tumor tissues compared with adjacent normal tissues. *Mex3a* expression as a categorical dependent variable was not associated with overall survival, and the overall survival of bladder urothelial carcinoma was associated with the group of age, cancer status, lymphatic vascular invasion, pathological stage, pathological size, and pathological lymph metastasis. The multivariable Cox model adjusted for the group of *mex3a* expression level, age, gender, tumor status, and pathological stage showed that only the age and cancer status groups were associated with the overall survival.

**Conclusion:**

*Mex3a* expression was not a poor prognostic factor of bladder urothelial carcinoma. Moreover, the expression levels of *mex3a* in the papillary type of bladder urothelial carcinoma were higher than those of the non-papillary type.

## INTRODUCTION

Bladder urothelial carcinoma (BLCA) is one of the most common tumors in humans and the second most frequently diagnosed genitourinary tumor [1, 2]. It has been reported that the occurrence and development of BLCA are multi-factorial, multi-stage, and involves multi-gene changes [3–5]. Although recent methods that employ novel technologies may be recognized as a promising option for bladder carcinoma treatment in the near future [6, 7], surgical operation is the primary method for treating bladder carcinoma, and chemotherapy is thought to be an effective adjunctive therapy to avoid the recurrence and metastasis of BLCA [8]. Considering that immunotherapy has become a treatment paradigm in bladder cancer and that drug resistance has increased, novel biomarkers are necessary and helpful to improve risk stratification and optimize the therapeutic choice of BLCA [9–11].

The gene *mex3a* is associated with tumor formation and may promote cell proliferation and tumor metastasis [12]. In this study, we did a thorough search for novel fusion transcripts in bladder cancer using RNA sequencing and sought to determine the effect of *mex3a* expression on the overall survival of BLCA. We analyzed the *mex3a* mRNA expression level in cases of BLCA and evaluated its prognostic value and whether *mex3a* could be a biomarker and a potential therapeutic target in high-risk BLCA.

## RESULTS

### Patient characteristics

Both clinical and gene expression data of the 412 primary tumors were acquired from the TCGA bladder cancer database, with data from 19 paired samples. Patients’ characteristics are shown in Table 1. Seventy-four percent of the cohort patients were men, and 26.2% were women. Approximately 79.4% were Caucasian, 5.6% were black of African-American descent, and 10.7% were Asian. Disease stages II, III, and IV corresponded to 32.3%, 34.2%, and 33.0% of the population, respectively. More than 50% of tumors (58%, n = 239) were of pathological n0, and 11.4%, 18.4%, and 1.9% of the tumors corresponded to pathologic stage n1, n2, and n3, respectively. In addition, 30.1%, 47.6%, 14.3% of the sample presented pathologic T2, T3, and T4 tumors, respectively, according to the American Joint Committee on Cancer (AJCC). Most tumor subtypes (66.5%, n=274) were non-papillary, and papillary tumors were detected in 32.3% of the sample (n=133). The outcomes of primary therapy were growing tumors (10.2%), complete remission (37.4%), solid tumors (5.6%), and partial remission (4.1%). Ninety-four percent (n=388) of the tumors were high-grade whereas 5.1% (n=21) were low-grade. More than 50% (57.0%, n=235) of the patients were tumor-free whereas 33.3% (n=137) had tumors. Thirty-two percent (n=132) of the patients did no present lymphatic vascular invasion whereas 37.1% (n=153) had a vascular invasion. The median follow-up for the subjects alive at last contact was 588 days (range of 0–4684 days).

**Table 1:** Characteristics of BLCA patients

Characteristic	Total	%
Gender	304	73.8
male	108	26.2
female		
Vital state		
alive	303	73.5
dead	109	26.5
Pathologic n		
N0	239	58.0
N1	47	11.4
N2	76	18.4
N3	8	1.9
Pathologic t		
T2	124	30.1
T3	196	47.6
T4	59	14.3
Pathologic stage		
stage ii	133	32.3
stage iii	141	34.3
stage iv	136	33.0
Cancer status		
with tumor	137	33.3
tumor free	235	57.0
Subtype		
non-papillary	274	66.5
papillary	133	32.3
lymphatic vascular invasion		
no	132	32.0
yes	153	37.1

### *Mex3a* expression in tumor tissues and adjacent normal tissues

**Figure 1:**
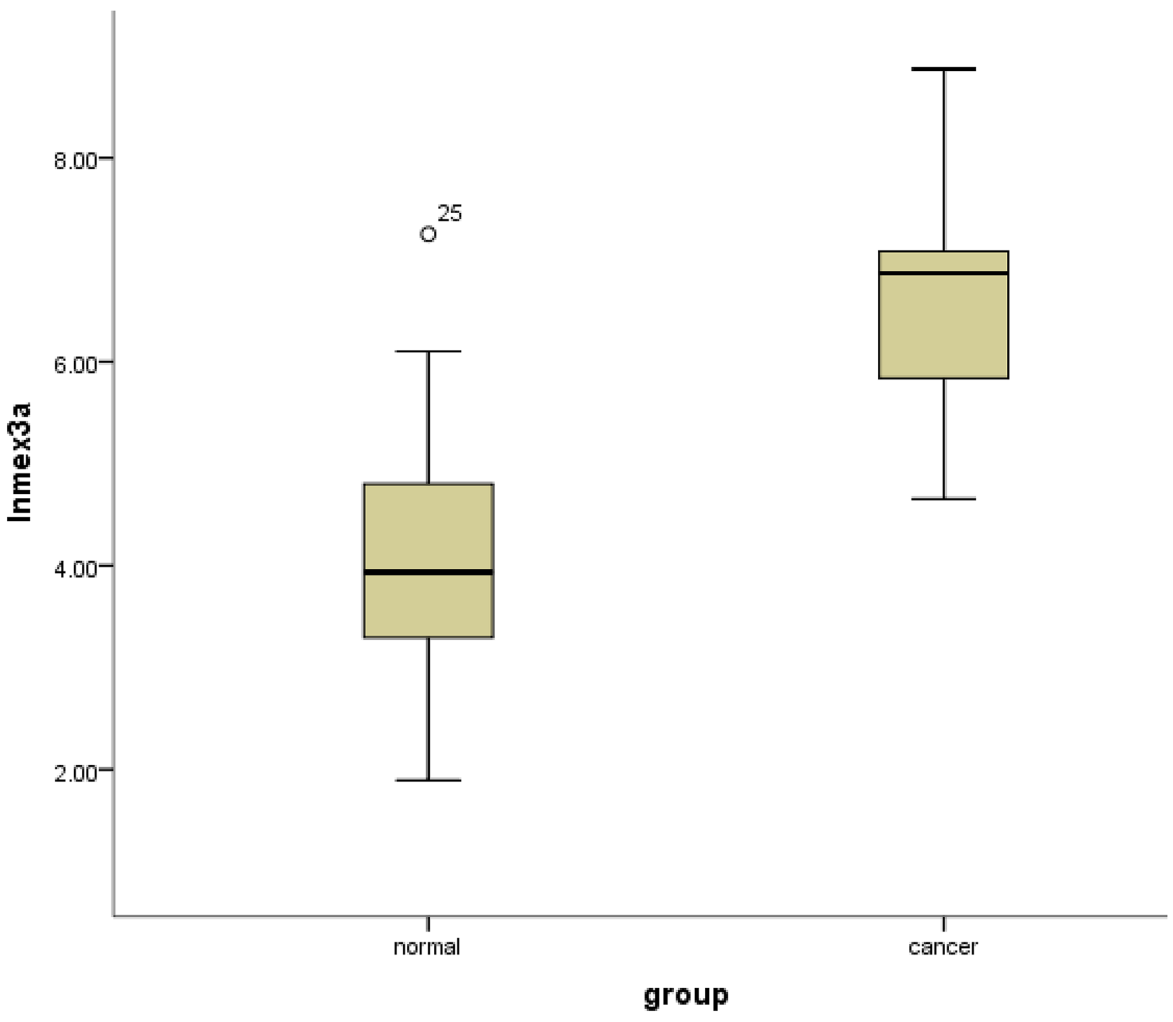
Box-plots shows the ln(Mex3a) value between cancer tissues and normal adjacent tissues

It was demonstrated that the mean normalized expression of *mex3a* was 1191.153 ± 1556.508 in cancer and 167.466 ± 323.095 in adjacent normal tissues. The normalized fold change of *mex3a* mRNA in cancer and normal tissues ranged from 0.89 to 151.22, and the mean fold change was 31.75 ± 46.75. The result of the independent sample *t*-test indicated that the level of *mex3a* expression was significantly higher in tumor tissues compared with adjacent normal tissues (*P*=0.008) (Figure 1).

### *Mex3a* expression and association with clinical variables

**Figure 2:**
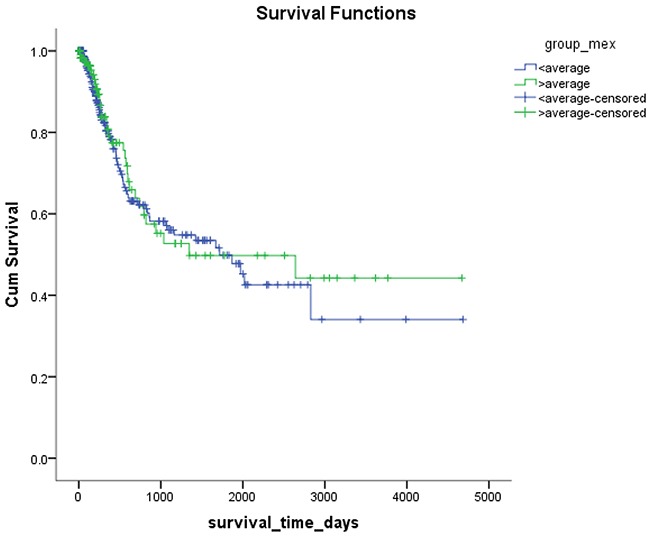
Kaplan-Meier plots of survival according to the high and low level of *mex3a*

**Figure 3:**
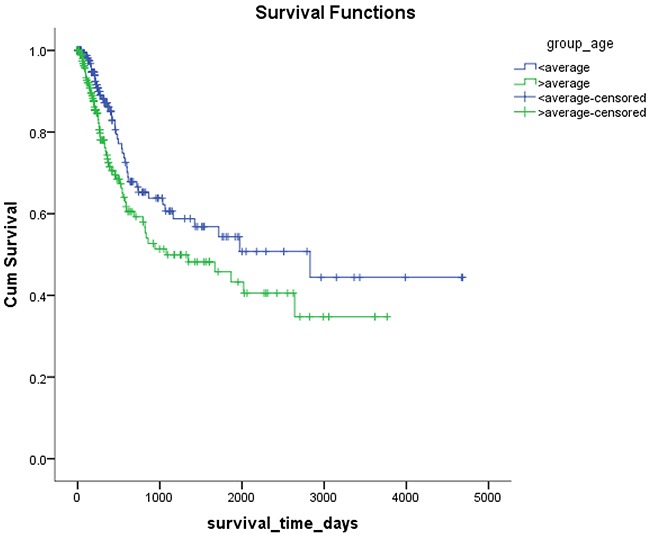
Kaplan-Meier survival plots according to the high and low age

**Figure 4:**
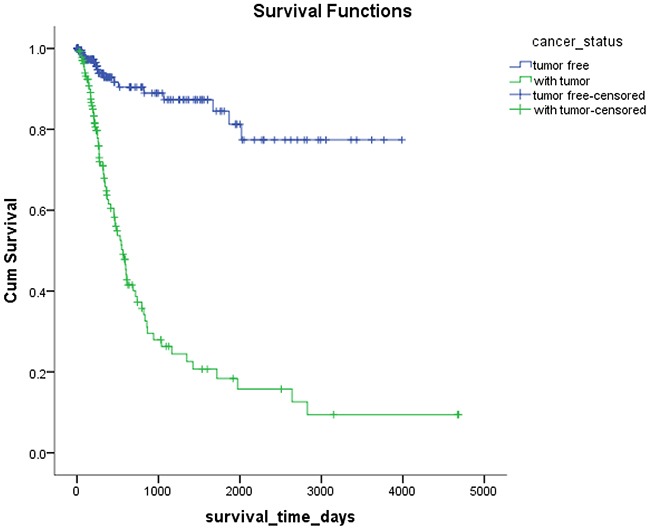
Kaplan-Meier survival plots according to the tumor status

**Figure 5:**
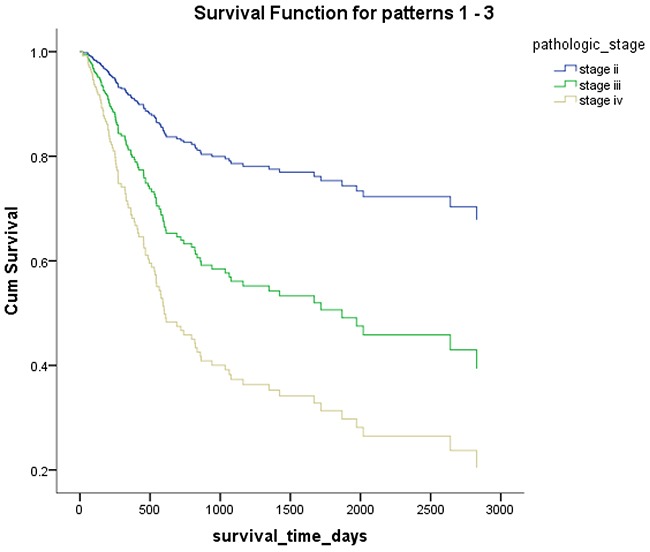
Overall survival according to the pathological stage

**Figure 6:**
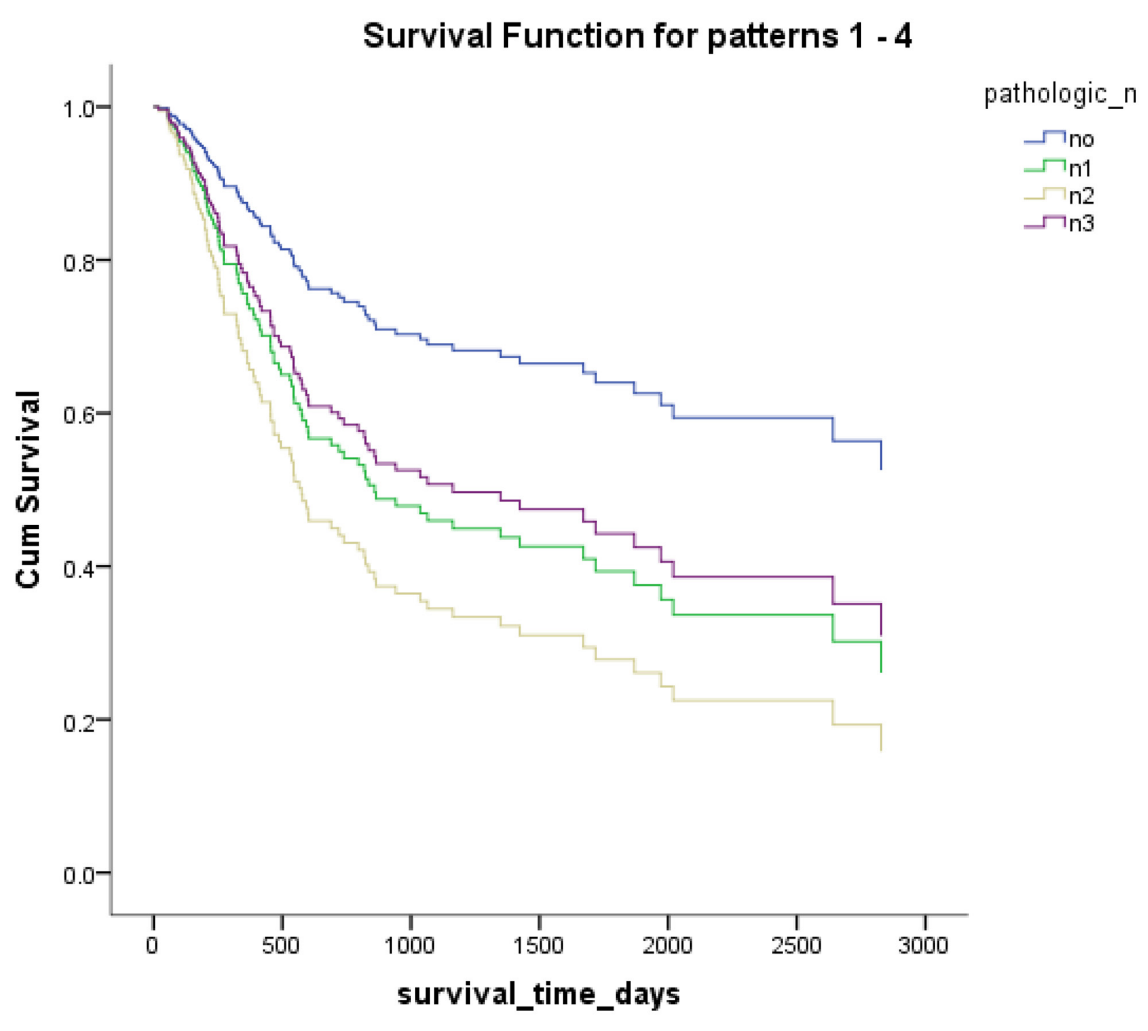
Overall survival according to pathological lymph node metastasis

**Figure 7:**
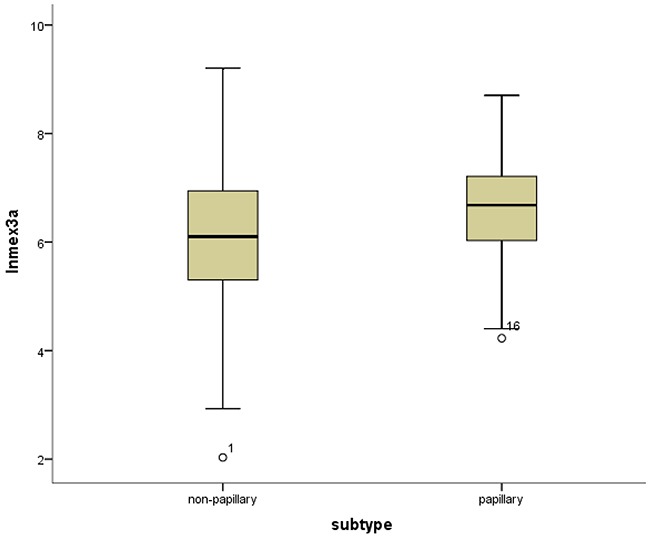
Box-plots shows the ln(Mex3a) value between the papillary and non-papillary subtypes

**Figure 8:**
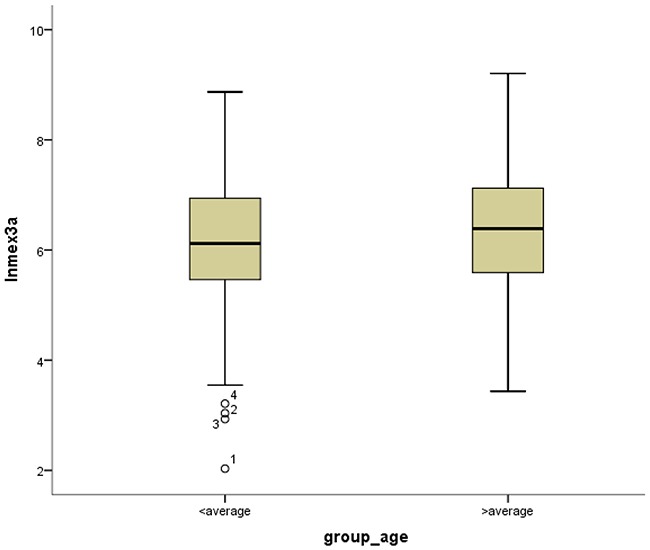
Box-plots shows ln(Mex3a) value between high and low age

*Mex3a* expression data for the 412 BLCA patients were acquired from the TCGA database. The median expression of *mex3a* as a categorical dependent variable in the univariate analysis was not associated with OS (*P*=0.673) (Figure 2). Other factors associated with OS are shown in Table 2. The older group first diagnosed with BLCA had poor OS (*P*=0.038, hazard ratio [HR] =1.505) (Figure 3), and this result agrees with that of a previous study [13] and with the mortality due to bladder cancer in China [14]. BLCA patients with tumor had a 7.9-fold higher risk of death than tumor-free patients (*P*<0.000, HR=7.883) (Figure 4). The increase in pathologic stage (Figure 5), pathological size, and pathological lymph metastasis (Figure 6) due to BLCA was associated with lower OS (*P*<0.000). The lymphatic vascular invasion was also associated with lower OS (*P*<0.000). The analysis of clinical-pathologic characteristics indicated that cancer subtype and gender were not associated with OS, (*P*=0.055 and *P*=0.513, respectively). The result of logistic regression analysis indicated that high *mex3a* expression was associated with the papillary type of BLCA (*P*=0.006, odds ratio [OR]=1.854) (Figure 7) and the older age group diagnosed with BLCA (*P*=0.027, OR=1.617) (Figure 8), but was not associated with tumor status (*P*=0.968) or pathologic stage (*P*=0.816).

**Table 2:** Cox regression univariate and multivariate analysis of OS for patients with BLCA

	Univariate analysis			Multivariate analysis		
	HR	95%CI	P value	HR	95%CI	P value
Group of age	1.505	1.024˜2.213	**.038**	2.653	1.430˜4.919	**.002**
Group of Mex	.914	.604˜1.385	.673	.606	.303˜1.210	.156
Gender	1.147	.761˜1.727	.513	1.495	.823˜2.716	.186
subtype	.621	.381˜1.010	.055	.938	.459˜1.915	.860
cancer status	7.883	4.638˜13.399	**<.000**	6.762	3.199˜14.290	**<.000**
Pathologic grade	1.938	1.495˜2.513	**<.000**	1.045	.505˜2.160	.906
Pathologic t	2.001	1.480˜2.706	**<.000**	1.284	.795˜2.074	.308
Pathologic n	1.631	1.319˜2.017	**<.000**	1.069	.654˜1.749	.790
Lymphatic vascular invasion	2.897	1.748˜4.800	**<.000**	1.639	.825˜3.254	.158

HR: hazard ratio; CI: Confidence interval.

### Survival outcomes and multivariate analysis

The multivariable Cox proportion hazards model adjusted for the *mex3a* expression group, and the group that included age, gender, cancer subtype, cancer status, pathologic stage, pathologic size, pathologic lymph metastasis, and lymphatic vascular invasion indicated that the older age group and the group with tumor were associated with OS (*P*=0.002, HR=2.653, *P*<0.000, HR=6.762, respectively). However, there were no significant differences in *mex3a* and OS between these groups.

## DISCUSSION

To date, a few studies have evaluated the effect of *mex3a* on tumor cells. *Mex3a* may be associated with tumors while has not yet seen its coverage on BLCA. A recent study from our research group indicated that *mex3a* could promote the proliferation of bladder urothelial cells (in press). Therefore, herein we assessed the correlation between *mex3a* expression and OS in BLCA.

The *mex-3* gene has a conserved region with 65–70 amino acids. This region interacts with RNA and contains two K-homology domains and a family of four human genes homologous to Mex-3 (hMex-3), which was first characterized in heterogeneous nuclear ribonucleoproteins [15]. The protein Mex-3 was initially believed to regulate the localization and activity of several maternal factors with transducer activity and polarity cue in the *Caenorhabditis elegans* embryo [16]. Buchet-Poyau K. [17] identified four potential human genes homologous to the *C. elegans* mex-3 gene (hMex-3A, -3B, -3C, and -3D) located on distinct chromosomes at positions 1q22, 15q25.2, 18q21.1, and19p13.3, respectively.

*Mex3a* is located at complement (156,072,013–156,081,998) and has 9986 base pairs. Jiang et al. [12] found that hMex-3D mRNA was ubiquitously expressed in all the cell lines and tissues tested by real-time polymerase chain reaction (RT-PCR) whereas the other three *hMex-3* genes were expressed at varying levels in different tissues. It was reported that the inactivation of *mex-3* by mutations could lead to embryonic death and defects in the anterior blastomere of the descendants. Jiang et al. [12] indicated that the knockout of gene *hMex3a* reduced the colony-forming ability of gastric cells in soft agar. Furthermore, transwell chamber and wound healing assays indicated that the knockout of *hMex3a* significantly affected the viability of cancer cells, and clinical correlation analysis indicated that the expression of *hMex3a* was significantly higher in cancer tissues than in normal tissues. Jiang et al. [12] demonstrated that *hMex3a* was involved in the regulation of tumorigenesis and that the aberrant activation of *mex3a* in human gastric cancer cells promoted cell proliferation and migration. Furthermore, the overexpression of *mex3a* was associated with the relapse of Wilms tumors [18]. Baumgart et al. [19] indicated that the expression of *mex3a* was similar to the expression of the mitotic marker proliferating cell nuclear antigen. A study [20] demonstrated that the overexpression of *mex3a* led to the downregulation of CDX2. Gross et al. [21] indicated that CDX2 inhibited cell growth and migration *in vitro* and the spread of colon tumor cells *in vivo*, indicating that the reduced expression of CDX2 caused by *mex3a* increased the progression of colorectal carcinomas induced by chemical substances.

The results of the independent sample *t*-test revealed significant differences in the expression of *mex3a* between cancer tissues and adjacent normal tissues and this result led us to evaluate whether *mex3a* was a biomarker of BLCA and whether *mex3a* could affect the overall survival of BLCA patients. Hence, the clinical data of 412 patients with BLCA were used to determine the factors associated with *mex3a* expression and whether these factors affected OS. However, the analysis of clinical data indicated that OS was not significantly different between the two groups of *mex3a* expression. The univariate Cox model showed that the high expression of *mex3a* decreased the risk of death by 8.5% (relative risk [RR]=0.914) and the multivariate Cox model showed that the high expression of *mex3a* decreased the risk of death by 39.4% (RR=0.606) although the differences were not significant. The decreased risk of death due to increased expression of *mex3a* provided discrepancies between the results of the univariate and multivariate Cox models. The result of logistic regression suggests that the cancer subtype strongly affects *mex3a* expression. Therefore, the effect of the cancer subtype on the expression of *mex3a* in the multivariate Cox model might be an important confounding factor. However, previous studies reported conflicting results [12, 19–21].

The univariate Cox model explored different factors separately, and the OS in BLCA was associated with age, cancer status, pathologic stage, pathologic size, pathologic lymph metastasis, and lymphatic vascular invasion. However, among the dummy variables, the absence of significant differences in pathologic lymph metastasis might be because the sample size was not enough for n3 and might lead to significant errors.

The results of the multivariate Cox model adjusted for the *mex3a* expression group, and the group that included age, gender, cancer subtype, cancer status, pathologic stage, pathologic size, pathologic lymph metastasis, and lymphatic vascular invasion indicated that only age and cancer status were associated with OS. The cancer status was divided into tumor status and tumor-free status. The former corresponded to the new tumor events after initial treatment whereas the latter corresponded to the status without new tumor after initial treatment. Our results indicated that higher age at diagnosis of BLCA had a 2.653-fold higher risk of death than lower age, and the tumor status had a 6.762-fold higher risk of death than the tumor-free status. The pathologic stage, pathologic size, pathologic lymph metastasis, and pathologic distant lymph metastasis as categorical dependent variables were associated with OS. However, when adjusted for other clinical–pathologic characteristics, these variables might not be associated with OS because of the strong confounding effects of the clinical-pathologic characteristics in the statistical analysis. For example, the pathologic stage was determined by the pathologic size, pathological lymph metastasis, and pathologic distant lymph metastasis according to tumor-node-metastasis (TNM) classification system. Therefore, the interaction between the factors produced a significant effect when pathologic staging and TNM staging were included in the Cox model simultaneously.

BLCA can be divided into invasive tumors and non-invasive tumors. Non-invasive BLCA is divided into papillary and flat types. The lack of infiltration into papillary structures is known as carcinoma in situ, with a high level of cytological characteristics. Papillary tumors include reactive hyperplasia, papilloma, low-grade potential, papillary urothelial proliferation of low malignant potential, and low-grade and high-grade papillary urinary tract epithelial cancers. Flat BLCA has a broad morphology, including reactive hyperplasia, precancerous lesions, and malignant lesions [22]. Most invasive cases of BLCA are confined to the lamina propria [22] and belong to the non-papillary type. In our patients with papillary bladder cancer, the expression of *mex3a* was increased, which might indicate that *mex3a* could be a classification marker for the BLCA subtypes. Significant differences were found between papillary and non-papillary BLCA tumors, and papillary tumors had a 1.854-fold higher likelihood of presenting a high expression of *mex3a* (*P*=0.006, OR=1.854). Different factors may affect age; therefore, although the mortality due to bladder cancer increased as age increased [14] and the older group diagnosed with BLCA had a 1.617-fold higher likelihood of presenting increased *mex3a* expression (*P*=0.027, OR=1.617), no correlation between *mex3a* expression and OS was observed.

Previous studies have shown that *mex3a* promoted cell division and metastasis and might be a hazard factor in gastric cancer [12] and Wilms tumors [18]. However, in this study, *mex3a* did not have a significant effect on the prognosis of BLCA, which might be because of dummy variables in the data from the TCGA database or missing data. However, other unknown factors might have a significant effect on *mex3a*. Therefore, the effects of *mex3a* on OS still need to be elucidated.

In conclusion, the expression level of *mex3a* was higher in cancer tissues compared with normal adjacent tissues. However, *mex3a* was not a poor prognostic factor of BLCA. Other factors, including increased age at diagnosis, tumor status, as well as the increase in pathologic stage, pathological size, and pathological lymph metastasis were associated with OS in BLCA. Moreover, *mex3a* expression was higher in the papillary type of BLCA than in the non-papillary type.

## MATERIALS AND METHODS

### Patients and methods

The clinical and RNA sequencing (RNA-Seq) expression data for bladder cancer patients were downloaded from the TCGA database (https://tcga-data.nci.nih.gov/tcga/tcgaDownload.jsp). The clinical data were matched with RNA-Seq data, which are used to study the transcriptome profiling and search for novel fusion genes [23]. A total of 412 BLCA samples were available in the TCGA database, and all these samples had corresponding RNA-Seq sequences, with a total of 19 paired samples. Clinical information was acquired from all samples. Clinical information of the BLCA patients was available from 19 paired samples, including age, gender, survival time, and tumor pathologic TNM staging. *Mex3a* expression was determined in both cancer and normal tissues and the expression levels were normalized.

The expression profile analysis was based on RNA-Seq data. The original data used for analysis were filtered and standardized before entering the subsequent statistical analysis. We only selected the most expressive transcripts for analysis, and the symbols with fewer than 50 original sequence reads were excluded from the analysis. The trimmed mean of M-values method was used to ensure data normality. This approach used the concept of relative expression between different samples to avoid the misalignment caused by the excessive absolute statistical method.

According to the patient barcode, the patients’ clinical information and *mex3a* expression level were matched, and RNA-Seq samples with 19 paired samples were also obtained. The *mex3a* group was classified into two groups (high and low expression) by the median *mex3a* expression value of 1007.2. The age group was classified into two groups by the median value of the patients’ age at first diagnosis of BLCA.

### Statistical analysis

Box-plots were used to express the differences in the *mex3a* expression between the groups. The univariate Cox analysis was used to evaluate the OS of BLCA with clinical-pathologic characteristics, respectively, as a categorical dependent variable. The multivariate Cox analysis was used to assess the effect of *mex3a* expression on the OS together with other clinical factors (the group that included age, cancer status, cancer subtype, gender, lymphatic vascular invasion, pathological size, pathological metastasis, and pathological stage as categorical dependent variables), and the survivorship curve was expressed to evaluate the OS among different clinical-pathologic characteristics. The univariate logistic regression analysis was used to determine the effects of distinct factors on *mex3a* among the dependent variables. An independent sample *t*-test was used to determine the differences in *mex3a* expression between tumor tissues and adjacent normal tissues according to data from the 19 paired samples. All data were analyzed with the software SPSS Statistics version 22.0. *P*<0.05 was considered statistically significant.

## Abbreviations

BLCA: Bladder Urothelial Carcinoma
TCGA: The Cancer Genome Atlas
OS: overall survival
AJCC: American Joint Committee on Cancer
CIS: carcinoma in situ
